# Clinical development of gene- and cell-based therapies: overview of the European landscape

**DOI:** 10.1038/mtm.2016.73

**Published:** 2016-11-30

**Authors:** Sofieke de Wilde, Henk-Jan Guchelaar, Maarten Laurens Zandvliet, Pauline Meij

**Affiliations:** 1 Department of Clinical Pharmacy and Toxicology, Leiden University Medical Center, Leiden, The Netherlands

## Abstract

In the last decade, many clinical trials with gene- and cell-based therapies were performed and increasing interest in the development was established by (national) authorities, academic developers, and commercial companies. However, until now only eight products have received marketing authorization (MA) approval. In this study, a comprehensive overview of the clinical development of gene- and cell-based therapies in Europe is presented, with a strong focus on product-technical aspects. Public data regarding clinical trials with gene- and cell-based therapies, obtained from the European Union (EU) clinical trial database (EudraCT) between 2004 and 2014 were analyzed, including product-technical variables as potential determinants affecting development. 198 unique gene and cell therapy products were identified, which were studied in 278 clinical trials, mostly in phase 1/2 trials and with cell therapies as major group. Furthermore, most products were manufactured from autologous starting material mostly manufactured from stem cells. The majority of the trials were sponsored by academia, whereas phase 3 trials mostly by large companies. Academia dominated early-stage development by mainly using bone marrow derived products and stem cells. Conversely, commercial sponsors were more actively pursuing *in vivo* gene therapy medicinal product development, and cell therapies derived from differentiated tissue in later-stage development.

## Introduction

Gene- and cell-based therapies hold great promise to provide novel treatment modalities for a wide range of severe diseases. Due to better insight into disease biology, cellular mechanisms, and rapidly evolving technology, development of regenerative and immunotherapeutic drugs is being pursued.^1^ In the European Union (EU), gene- and cell-based therapies are defined as advanced therapy medicinal products.^2^ The majority of these therapies find their origin in academic research settings,^3^ and small companies have increasingly been involved. Recently, major pharmaceutical companies have invested in gene- and cell-based therapy development as well in both the EU and in the United States.^4,5^

Current interest in the (pre)clinical development of these promising products is illustrated by a substantial amount of 12,000 publications in regenerative medicine and tissue engineered products in PubMed between 2012 and 2015 and 15,618 on advanced therapy medicinal products in the last 5 years (PubMed search: “advanced therapy medicinal products OR advanced therapy medicinal products OR gene therapy product OR cell therapy medicinal products OR Tissue engineered products” search performed on 14 August 2016).^6^ However, despite this impressive scientific output, only few of the products have received marketing authorization approval, with the result that there is only limited benefit for patients.^6–8^ Inherent product-technical aspects may have hampered clinical development, such as the availability of human starting material, or small-scale or even fully personalized manufacturing strategies.^9^ The fact that this is a relatively new field, which used to rely on the academic institutions, also might have impacted this outcome. To be able to explain this disappointing outcome regarding the marketing authorization approvals, first more insight is needed into the clinical development of these products.

Up to now, several attempts were made to analyze the clinical development of gene- and cell-based therapies by generating overviews of clinical trials. Maciulaitis *et al.*, generated an overview of the European gene and cell therapy clinical trials based on the European pharmacovigilance database EudraCT in the period of 2004 until 2010.^10^ More recently, a global overview of cell therapy clinical trials was generated by Bersenev.^11^ However, this latter overview was not peer-reviewed, and was incomplete due to exclusion of gene and tissue engineered therapies.^11^ Despite providing an overview of the field, both analytical studies provided limited information on the product-technical aspects, which are of vital importance to identify gene- and cell therapy-specific hurdles in clinical development.

To be able to identify the reasons for failure of products to achieve marketing authorization in the future, it is important to first have a comprehensive overview of the clinical development of the gene- and cell-based therapy products. This overview is also focused on product-technical features, such as product subtype and starting material sources. In this study, we aim to systematically analyze all EU clinical trials performed in the last decade. Furthermore, our second goal is to establish which factors may influence product design and clinical development.

## Results

### Overview of gene- and cell-based therapy clinical trials

**Figure 1:**
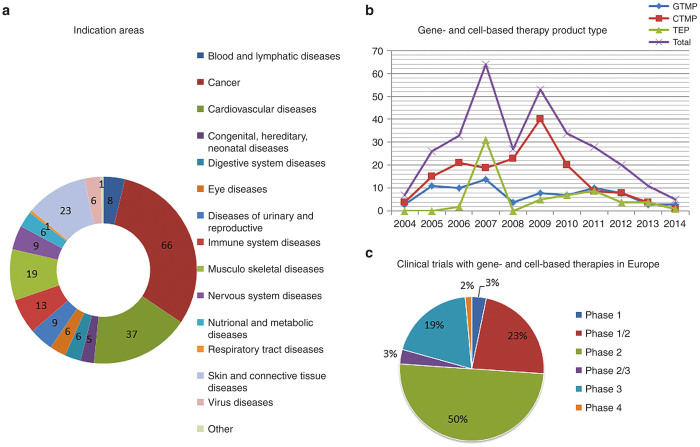
Gene- and cell-based therapy landscape. CTMP, cell therapy medicinal product; GTMP, gene therapy medicinal product; SME, small and medium-sized enterprise; TEP, tissue-engineered product. (**a**) Distribution of the indication areas for the gene- and cell-based therapy, showing absolute numbers derived from the public domain of the EudraCT database*; (**b**) Absolute numbers of clinical trials with the of gene- and cell-based therapy product types per year; (**c**) Numbers (represented in percentages, *n* = 278) of different clinical trials study phases performed with gene- and cell-based therapy products. * Since phase 1 clinical trials are not in the public domain of EudraCT and processing of trials in EudraCT can be delayed, clinical trials may be missing.

From public domain of the EudraCT database (since phase 1 clinical trials are not in the public domain of EudraCT and processing of trials in EudraCT can be delayed, clinical trials may be missing), 278 gene- and cell-based therapy clinical trials were identified over the period 2004 until 2014, corresponding with 198 unique gene- and cell-based therapy products. The majority of these products was tested in one clinical trial only (75% from total *n* = 198), while the remaining part was tested in two (17%) or in three or more studies (8%). Half of the products, tested in more than one clinical trial, reached a next clinical trial phase, whereas the other half remained in the same clinical trial phase. The dominant indication areas were cancer (31%), cardiovascular diseases (17%), and skin and connective tissue diseases (11%) (Figure 1a; total *n* = 278).

The total number of clinical trials increased to maximum numbers between 2007 and 2009, after which the numbers in the database showed a decrease towards 2014 (Figure 1b). Half of the clinical trials were phase 2, while 23 and 19% were phase 1/2 or phase 3 trials, respectively (Figure 1c; total *n* = 278). Since data about phase 1 clinical trials are not available in the public domain of the database, these trials were not included in the analysis. Most trials were performed using cell therapy products (53%).

### Country-specific variations in clinical gene- and cell-based therapy development

**Figure 2:**
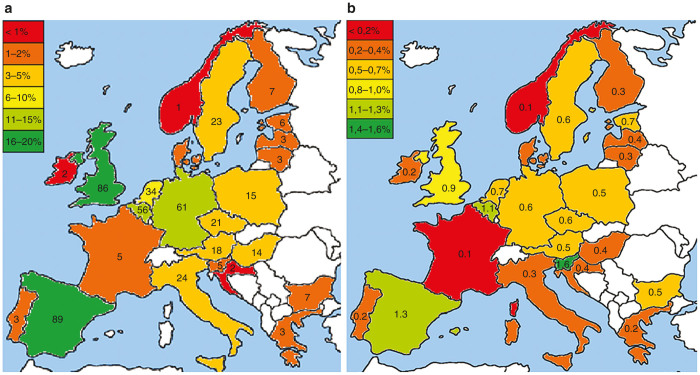
Country-specific variations in gene- and cell-based therapy development. (**a**) Percentage of clinical trials with gene- and cell-based therapies per country derived from the public domain of the EudraCT database* depicted in color and absolute numbers of clinical trials performed per country (written in country). (**b**) Ratio of the number of clinical trials with gene- and cell-based therapy products performed in a country per the total amount of clinical trials in that country derived from the public domain of the EudraCT database. The white colored countries did not perform any studies with gene- and cell-based therapy products or do not belong to the European Union. (European map adjusted from http://www.youreuropemap.com/). * Since phase 1 clinical trials are not in the public domain of EudraCT and processing of trials in EudraCT can be delayed, clinical trials may be missing.

More than half of the clinical trials (65%) were performed in only five of the EU countries (Figure 2a; *n* = 499). Most trials were performed in Spain (18%), investigating predominantly cell therapy products (71%), and in the United Kingdom (17%), where half of the trials were executed with gene therapy products (Supplementary Figure S1). The other three countries in which there is a clear high clinical activity were Germany (12%), Belgium (11%), and The Netherlands (7%). To further specify these figures, trials with tissue engineered products were mostly performed in Germany (17%). Gene and cell therapy trials contributed less than 1% of the overall clinical trial numbers in the EU (range 0.1–1.6% per country) (Figure 2b).

### Sponsor variations in clinical gene- and cell-based therapy development

**Figure 3:**
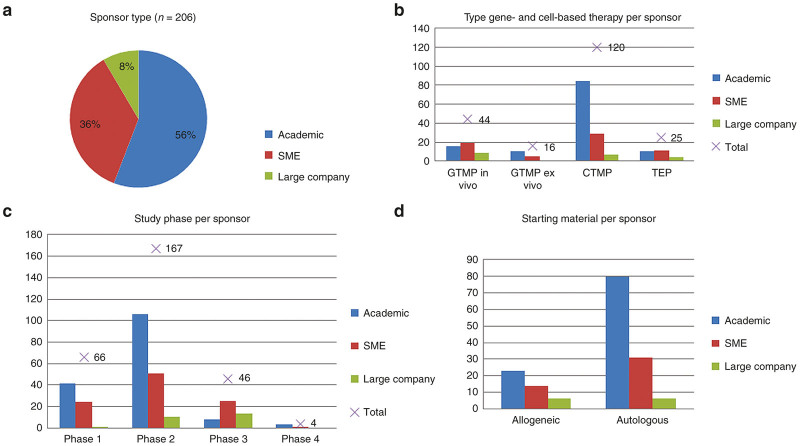
Sponsor type variations in gene- and cell-based therapy development. CTMP, cell therapy medicinal product; GTMP, gene therapy medicinal product; SME, small- and medium-sized enterprise; TEP, tissue-engineered product. (**a**) Distribution of the different sponsors that performed clinical trials with gene- and cell-based therapies. (**b**) Absolute numbers of clinical trials performed with gene- and cell-based therapy types per sponsor and the total absolute number of clinical trials with the different gene- and cell-based therapy types. (**c**) Absolute numbers of clinical trials in different clinical phases per sponsor and the total number of clinical trials per clinical phase. (**d**) Starting material per sponsor type.

Sponsor types identified were academia, small- and medium-sized enterprise (SMEs) and large pharmaceutical companies. More than half of the trials was sponsored by academic institutes (56%), while 36% of the trials was sponsored by SMEs and only 8% by large companies (Figure 3a; total *n* = 206).

Most clinical trials sponsored by academic institutes and SMEs, were conducted with cell therapy products (70%; *n* = 84 and 45%; *n* = 29 respectively), and most of these were phase 2 trials (67%; *n* = 106 and 51%; *n* = 51 respectively) (Figure 3b,c and Supplementary Figures S2 and S3). In contrast, large companies focused on clinical trials with *in vivo* gene therapy products (43%; *n* = 9) and sponsored more than half of the phase 3 trials (54%; *n* = 13). Furthermore, another interesting finding is that single center and national trials were predominantly sponsored by academic institutes (68%), while nearly all multinational trials were sponsored by SMEs or large companies (94%). Remarkably, for large companies, skin and connective tissue diseases was a relatively prominent indication (26%) (Supplementary Figure S4) (*n* = 219).

### Product-technical variation in clinical gene- and cell-based therapy development

Next, we systematically analyzed the product-technical characteristics of gene- and cell-based therapy products in clinical development within the EU in more detail.

The major product subtypes were *in vivo* gene therapy products (22%; *n* = 44), stem cell products (19%; *n* = 38) and mesenchymal stromal cells (16%; *n* = 32). Whereas most of the stem cell products were developed for treating cardiovascular diseases (51%; *n* = 29) and differentiated tissue cell products for skin and connective tissue diseases (45%; *n* = 20) (Supplementary Figure S5), most of the dendritic cell products (89%; *n* = 32), lymphocyte-based products (82%; *n* = 9), and gene therapy products (52%; *n* = 43) were designed for cancer treatment. Academic sponsors (total *n* = 116) mainly performed clinical trials with stem cells, dendritic cells, and mesenchymal stromal cells as largest subtypes (24, 20, and 20%, respectively), while both SMEs (total *n* = 61) and large companies (total *n* = 20) performed clinical trials with *in vivo* gene therapy products as the largest group (31 and 45%, respectively) (Supplementary Figure S6).

The majority of the products were produced from autologous starting material (73%; *n* = 117), of which most often stem cell products were manufactured (31%; *n* = 49) (Figure 3d and (Supplementary Figures S7 and S8). Although in general differentiated tissue was most frequently used as source for starting material (31%; *n* = 68), autologous products were mostly generated using bone marrow-derived starting materials (33%; *n* = 52) (Supplementary Figure S9). Academic sponsors and SMEs mainly used autologous starting material for the manufacturing of their products (78%; *n* = 80 and 69%; *n* = 31 respectively), which were most frequently derived from bone marrow in case of academic sponsored studies (29%; *n* = 36) and most frequently from differentiated tissue when sponsored by SMEs (30%, *n* = 19) (Supplementary Figures S7 and S10). Furthermore, autologous starting material is used most often for the production of cancer therapies (31%; *n* = 48) (Supplementary Figure S11). In addition, cancer therapy products were generated from peripheral blood in approximately half of these cases (49%; *n* = 45), whereas the majority of the cardiovascular disease therapy products were generated from autologous bone marrow (63%; *n* = 30) (Supplementary Figure S12).

Most of the allogeneic products were manufactured toward differentiated tissue cells and mesenchymal stromal cells (31%; *n* = 19 each). Half of the products generated by large companies were allogeneic products and were mostly derived from differentiated tissues (23%; *n* = 5). Furthermore, it seems that these allogeneic products were mainly manufactured for treatment of skin and connective tissue diseases (24%; *n* = 15).

## Discussion

This study provides a detailed and comprehensive overview of all clinical trials performed with gene- and cell-based therapy products in Europe over the last decade, and represents the current status of gene- and cell-based therapy development in Europe. The majority of trials were performed by academic sponsors, most often using autologous starting material and they mostly manufactured stem cell products. Commercial companies performed most studies with *in vivo* gene therapy products and were nearly always involved in later stage phase 3 clinical trials. Only 11% of these products proceeded toward a clinical trial of the next phase clinical trial, suggesting that clinical development of most products hampered.

The number of clinical trials increased in the period from 2004 to 2010, including two peaks in 2007 and 2009, and thereafter showed a decrease. The first peak, with tissue-engineered products as the major group, might be explained by the fact that the EU directive 2001/83/EC, which defines the gene- and cell-based therapy products, was renewed in 2007 adopting tissue-engineered products.^12^ The other peak in 2009 might be due to the specific regulation for gene- and cell-based therapies (EC) no 1394/2007 that came into force on 30 December 2008. Most therapies were designed for the treatment of cancer and cardiovascular diseases, which can be explained by the fact that these cause most deaths globally.^13^ Therefore, improved clinical development of gene- and cell-based therapies may eventually yield clinical benefit for those diseases in particular where there is a high unmet medical need.

Spain and the United Kingdom are the countries in which most of the clinical trials in Europe were performed. Interestingly, gene- and cell-based therapy development has been stimulated in these countries by specific initiatives for facilitating financial resources, knowledge and (design of) centralized good manufacturing practice facilities.^14,15^ As such, infrastructure appears to have a relevant impact on further development of these promising therapies, regional and national public stakeholders are encouraged to consider similar approaches.

The majority of the products were sponsored by academia, whereas 61% of the clinical trials with conventional medicinal products originated from commercial companies in the European Economic Area (EEA).^16^ From earlier research, the (regulatory) route toward marketing authorization (MA) was found to comprise many hurdles for academic research groups.^9^ Indeed, all gene- and cell-based therapies that received MA approval were sponsored (in later stages of development) by a commercial party. In our database, it is shown that nearly all phase 3 trials were performed by commercial sponsors, supposing that they have appropriate financial resources and (regulatory) knowledge.

The slight increase (40% to 44%) in commercially sponsored trials from 2010 to 2014, including an increase from 2 to 8% of the large companies involved, suggest increasing interest in gene- and cell-based therapy development by the commercial (large company) sector.^10^ In comparison, commercial sponsors developed more off-the shelf products, such as allogeneic products and *in vivo* gene therapy products, compared to academic sponsors. This is to be expected, as late stage off-the-shelf product development is easier and less expensive, because it entails large scale manufacturing and efficient logistics, when compared to personalized autologous or donor-matched manufacturing. However, all cell-based therapies (4) with MA approval in Europe were generated from autologous starting material (Chondrocelect, Holoclar, MACI (currently suspended), and Provenge (retracted in 2015)) and had a commercial sponsor, indicating that personalized manufacturing did not hamper their (commercial) development. Due to the potential efficacy and safety of fully personalized therapies, the authors believe that (large) commercial sponsors become more interested in the development of these gene- and cell-based therapies toward MA, as seen in the examples of CTL019 and Strimvelis.^4,5^ The growth of investments from large pharmaceutical companies in gene-and cell-based therapy development, is a clear indication of the fact that there is an increasing involvement of the commercial sector in this young field of medicinal products.^4,5^ We expect that this trend of more involvement from commercial parties will continue to date. In particular, academic-commercial partnerships may be very efficient to drive the best cell- and gene-based products from early academic research toward proper late-stage clinical development. The number of Committee for Advanced Therapies (CAT) classifications and scientific advice applications for gene- and cell-based therapy products has increased over the last years. A proactive attitude of regulators, as recommended by Maciulaitis *et al.*, may have contributed to this increase.^10^ The involvement of and regulatory advice from the central expertise of the EMA CAT is expected to support increasing numbers of MAs over the next years.^8,17,18^ Since gene- and cell-based therapies are developed globally, the question is whether their development results more often in MA approval in other jurisdictions. In the United States, Canada, and Japan, whose systems for protection of the public health are corresponding to the European system,^19^ six, one and two gene- and cell-based therapies, respectively received MA approval. This supports the idea, that this lack in further development of these therapies is global. In contrary, South Korea has 18 gene- and cell-based therapy products with MA approval,^19^ suggesting that their specific regulation for these therapies supports development to MA approval.

The largest product group manufactured from autologous starting material was stem cell products. In academic institutes, the autologous products were often derived from bone marrow. This may be explained by the fact that these stem cell products have similarity with hematopoietic stem cells which have already been in use for bone marrow transplantation in these institutes for decades. Furthermore, the autologous products were mostly manufactured for cancer therapy, which might be due to the durable immune stimulatory potential of personalized treatment.

This study provides a comprehensive overview of gene- and cell-based therapy product development in the EU. However, since we only had access to the public domain of EudraCT, the phase 1 clinical trials were not included, resulting in an underestimation of the total number of clinical trials and products. Moreover, some clinical trials may not have been included in the public database due to delayed processing of clinical trials into EudraCT.

In order to stimulate clinical development of autologous products toward MA, academic, and commercial parties may collaborate efficiently in public-private partnerships. Furthermore, public stakeholders should consider stimulating efficient development of gene- and cell-based therapies toward patient benefit, *e.g.*, via regional or national initiatives.

Based on these results, future studies are necessary to further identify factors associated with success and failure of gene- and cell-based therapy development. These insights may help stakeholders to more effectively use their resources to increase availablity of safe and effective gene- and cell-based therapies.

## Materials and Methods

### Search strategy EudraCT

On 13 February 2015, a web-based data extraction was executed from the web portal, www.clinicaltrialsregister.eu, of the EudraCT database for pharmacovigilance based on EU Directive 2001/20/EC, using the following queries:

- Public access data: Yes
- Field “Advanced Therapy IMP” OR field “Somatic cell therapy medicinal product” OR field “Gene therapy medicinal product” OR field “Tissue Engineered Product” has to be answered with “YES”

The following data fields, from the EudraCT form, were extracted:

{A.1-Member State submission} {A.2-EudraCT number} {A.3-Full title trial} {A.3.2.-Name of abbreviated title} {A.7-Is the trial part of a PIP?} {A.8-EMEA decision number PIP}B.3.1/2-Status Sponsor} {B.5-Functional name contact point sponsor} {B.5.3.1 t/m B.5.3.6-Address contact point sponsor} {D.1.1-IMP number} {D.1.2-IMP being tested} {D.1.3-IMP used as a comparator} {D.2.1-Has this IMP to be used in the trial a marketing authorisation?} {D.2.5-Orphan drug designation?} {D.2.5.1-Orphan drug designation number} {D.3.1-Product name} {D.3.2-Product code} {D.3.4-Pharmaceutical form} {D.3.7-Route of administration} {D.3.8-(proposed) INN} {D.3.11.2-Active substance of biological/biotechnological origin (other than ATIMP)?} {D.3.11.3-TIMP?} {D.3.11.3.1- sCTMP?} {GTMP?} {D.3.11.3.3-TEP?} {D.3.11.3.4-Combination ATIMP} {D.3.11.3.5-CAT classification?} {3.11.3.5.1-ATIMP classification number} {D.3.12-Mode of action} {E.1.1-Medical condition(s) to be investigated} {E.1.1.2-Therapeutic area} {E.1.2-MedRA version, level, term, number} {E.1.3-Rare disease?} {E.2.1-Main objective} {E.2.2-Secondary objective} {E.5.1-Primary endpoint} {E.5.2-Secondary endpoint} {E.6.3-Therapy} {E.6.4-Safety} {E.6.5-Efficacy} {E.6.9-Dose Response} {E.7.1-Phase 1} {7.1.1-First administration to humans} {E.7.1.3-Other} {E.7.2-Phase 2} {E.7.3-Phase 3} {E.7.4-Phase 4} {E.8.1-Controlled trial} {E.8.1.1-Randomised trial} {E.8.1.2-Open trial} {E.8.1.3-Single blind trial} {E.8.1.4-Double blind trial} {E.8.1.5-Parallel group} {E.8.1.6-Cross-over} {E.8.2.1-Control group: Other medicinal product} {E.8.2.2-Control group: placebo} {E.8.2.3- Control group: other} {E.8.3-Single site in member State} {E.8.4-Multiple sites in Member State} {E.8.4.1-Numbers of sites} {E.8.5-Multiple Member States} {E.8.5.1-Number of member states in EEA} {E.8.6.1-Trial involving sites within and outside EEA} {E.8.9-Initial estimate of duration trial} {F.1.1-Age trial: Less than 18 years} {F.3.1-Healthy volunteers as subjects} {F.3.2- Patients as subjects} {P – End of trial}.

### Selection

The selection based on the queries (see “Search strategy EudraCT”), resulted in 754 trial data records, containing all data fields in a Microsoft Excel 2010 file. Two authors independently classified the products as being a gene therapy product, a cell therapy product or a tissue engineered product, according to the article 17 of regulation (EC) No. 1394/2007 (ref. 17). In case of disagreement (29 cases), a third author independently performed the classification, after which the three authors agreed on the classification by consensus. In case a product received a scientific recommendation on classification of the product by the EMA CAT,^20^ this classification was used. The gene therapy products were divided into two subtypes: *in vivo* gene therapy products, meaning that genetic material is administered directly into the patient, and *ex vivo* gene therapy products, when autologous or allogeneic cells are genetically modified *ex vivo* and (re)administered into the patient. If needed, additional information was collected from websites with information about the clinical trials (*e.g.*, Clinicaltrial.gov), PubMed, and websites from the sponsors.

**Figure 4:**
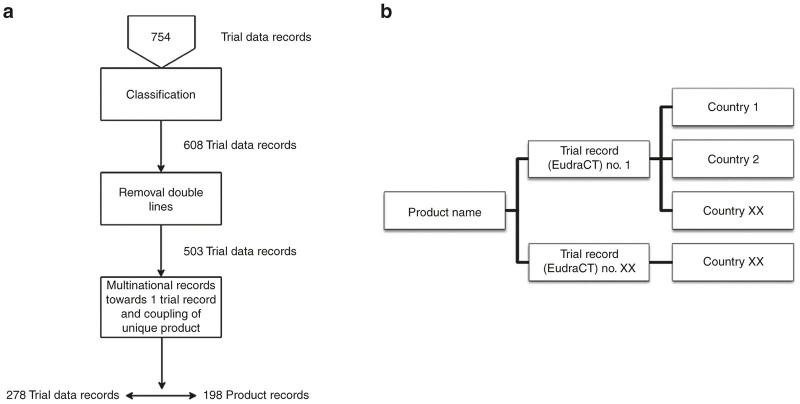
Database record selection and arrangement. (**a**) A schematic overview of the EudraCT data record selection. Trial data records from the EudraCT queries were reduced towards product records with corresponding clinical trials. (**b**) A schematic overview on how the database ProMISe was built, by coupling of clinical trials to the product and coupling of the countries in which the clinical trials have been performed.

146 trial data records did not meet the gene- and cell-based therapy product classification and were deleted. Subsequently, identical trial data records were deleted (105 records) leaving 503 trial data records for analysis. Coupling of the multicenter (multinational) performed trial data records towards one trial data record, corresponding with the same product record, was done based on: product name, product subtype, and sponsor name and resulted in 278 trial data records, corresponding with 198 unique product records (Figure 4a).

To ensure reliability, security, (error) sensitivity with the trial data records and the product data records, a web-based database, Project Manager Internet Server (ProMISe) was used.^21^ All data and product records were exported from the Microsoft Excel file towards ProMISe in a specific structure (Figure 4b). Product name (product record) was used as the main record in the ProMISe database, and each product record was linked to one or more trial records (EudraCT numbers). The first four numbers of the EudraCT number correspond with the year in which the EudraCT number was assigned. These year numbers were used for annual overviews.

Classification of the sponsor type was done by one author, based on the SME criteria from regulation (EC) No 726/2004: a company was considered as an SME when <250 staff headcounts AND Turnover ≤ €50 m OR balance sheet total ≤ € 43 m. If a commercial company did not meet the characteristics for a SME, it was classified as being a large company.

Product-technical specifications: product subtypes and starting material sources were classified by three authors independently (Table 1). In case of any disagreement, the product was discussed by the three authors to reach consensus about the product-technical specifications.

### Analysis

The data in ProMISe was analyzed in both ProMISe and Microsoft Access 2010. By generating frequency tables of different variables, cross tables, overviews of the gene- and cell-based therapy development in Europe were created. The following variables were analyzed: gene- and cell-based therapy product type, year of achieving EudraCT number, indication area, country, sponsor type, product subtype, starting material (autologous or allogeneic), and starting material source. See Table 1 for classification of starting material source and product subtype.

## Figures and Tables

**Table 1:** Gene- and cell-based therapy product type starting material source and product subtypes

*Gene- and cell-based therapy product type*	*Starting material source*	*Product subtype*
Cell therapy medicinal product (CTMP)	Differentiated tissue	Dendritic cells
Gene therapy medicinal product (GTMP)	Blood	Differentiated tissue cells
Tissue engineered product (TEP)	Bone marrow	GTMP—*ex vivo*
	Tumour tissue	GTMP—*in vivo*
	Other	Lymphocytes
		Mesenchymal stromal cells
		Stem cells
		Other

## Notes

The authors declared no conflict of interest.

The last two authors contributed equally to this work.
