# Supplementary material for: Clinical development of gene- and cell-based therapies: overview of the European landscape
Source: Mol Ther Methods Clin Dev. 2016 Nov 30;3:16073–. doi: 10.1038/mtm.2016.73 (PMC5130079; doi:10.1038/mtm.2016.73)

Figure S1. Country-specific clinical trials.

CTMP = cell therapy medicinal product; GTMP = gene therapy medicinal product; TEP = tissue-engineered product; UK = United Kingdom (a) Absolute numbers of the clinical trial phases performed in the five main countries. (b) Absolute numbers of clinical trials per gene and cell based product type in the five main countries.

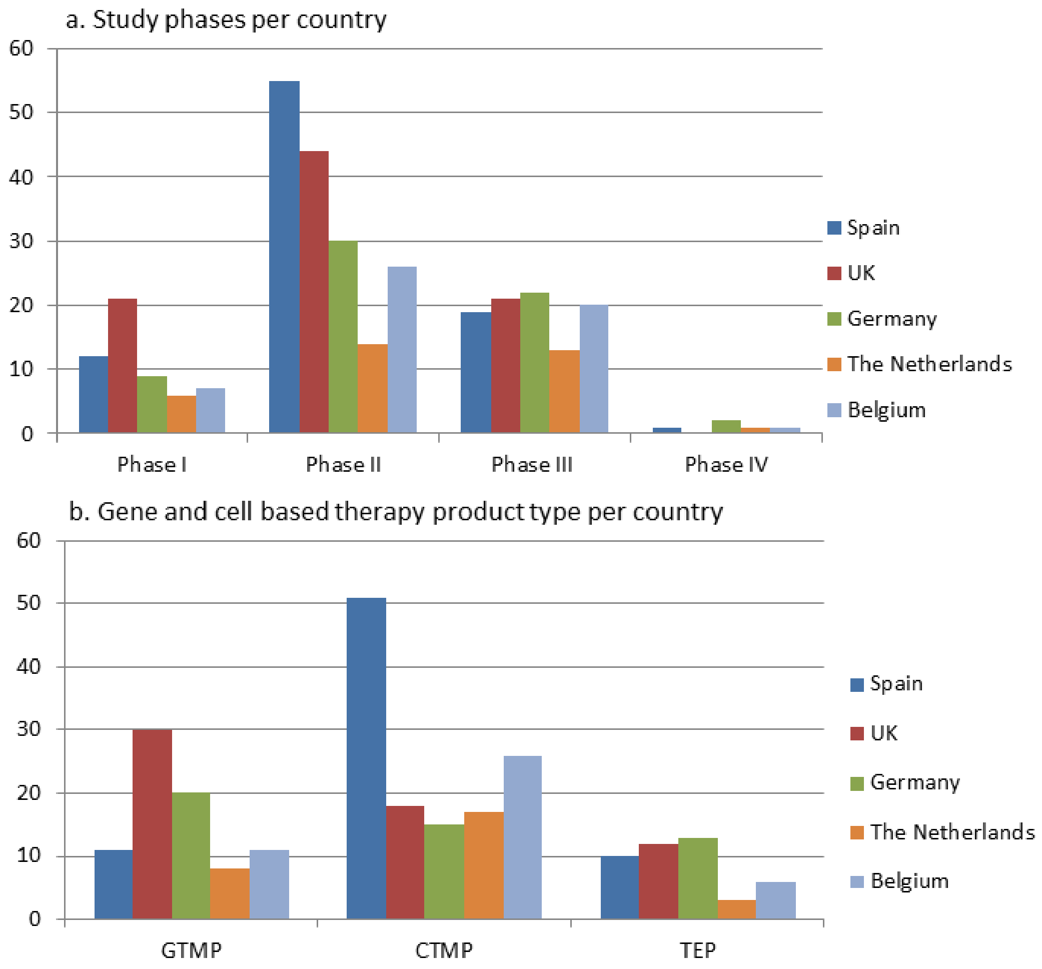

Figure S2. Gene and cell based therapy per sponsor.

CTMP = cell therapy medicinal product; GTMP = gene therapy medicinal product; SME = small and medium-sized enterprise; TEP = tissue-engineered product. The number of gene and cell based product type per sponsors (represented in percentages).

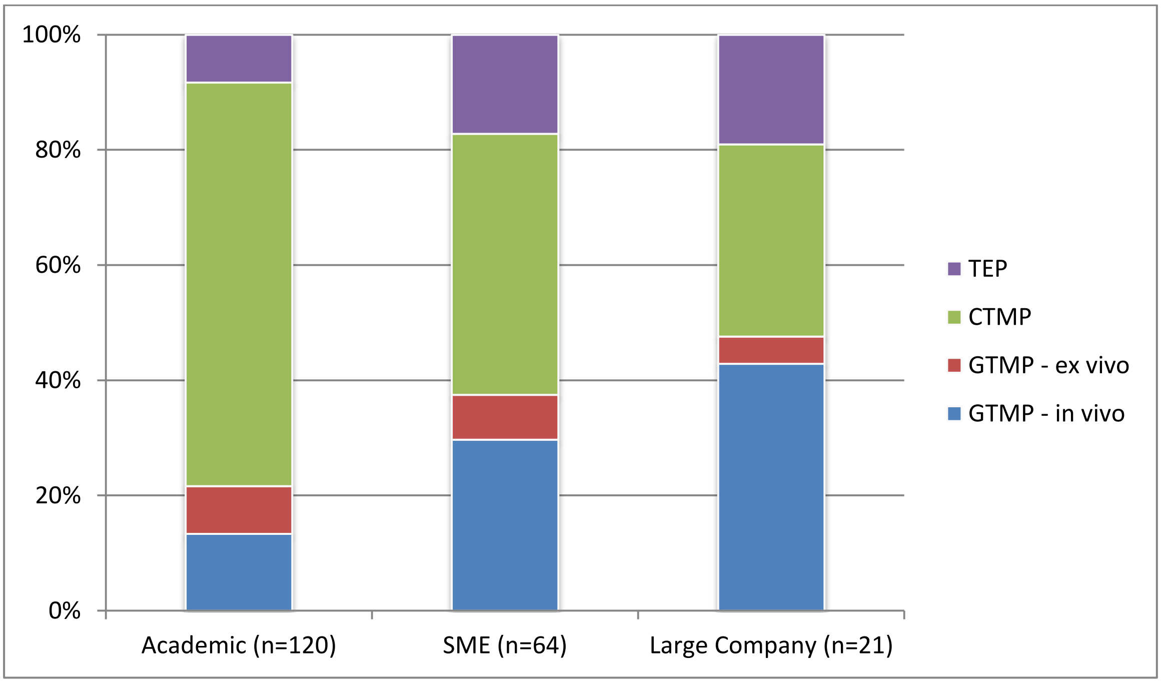

Figure S3. Gene and cell based therapy clinical phase per sponsor.

SME = small and medium-sized enterprise. The number of clinical trial phases performed per sponsor type (represented in percentages).

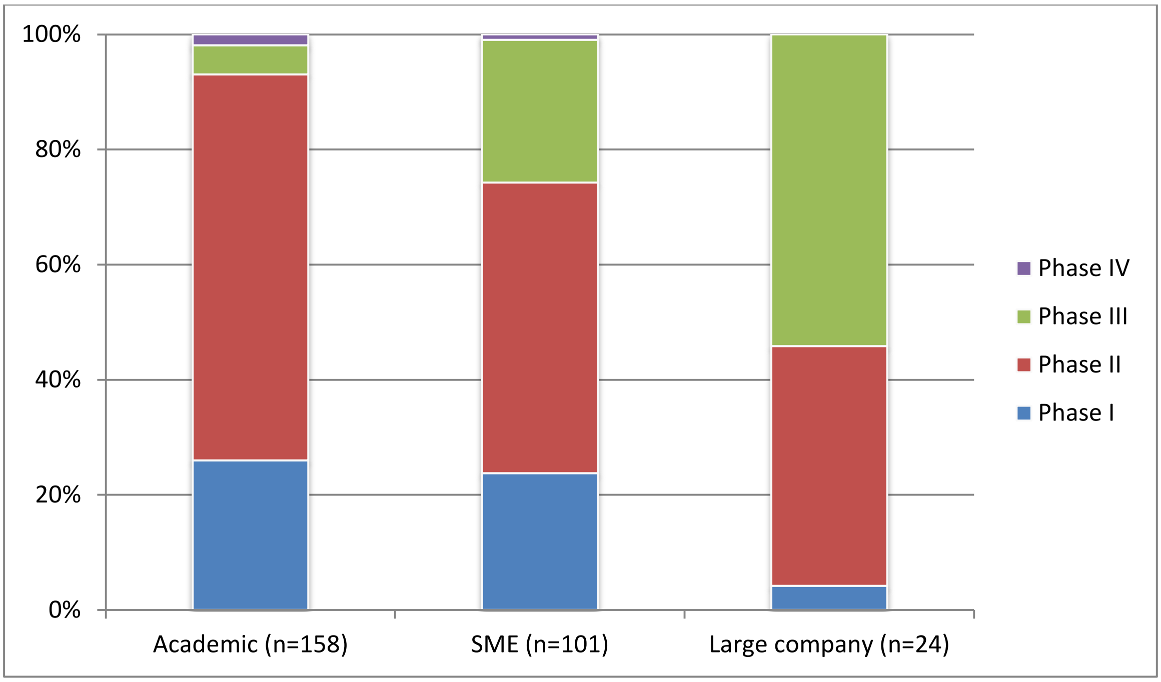

Figure S4. Indication area per sponsor.

SME = small and medium-sized enterprise. Distribution of the different indication areas in which clinical trials were performed per sponsor.

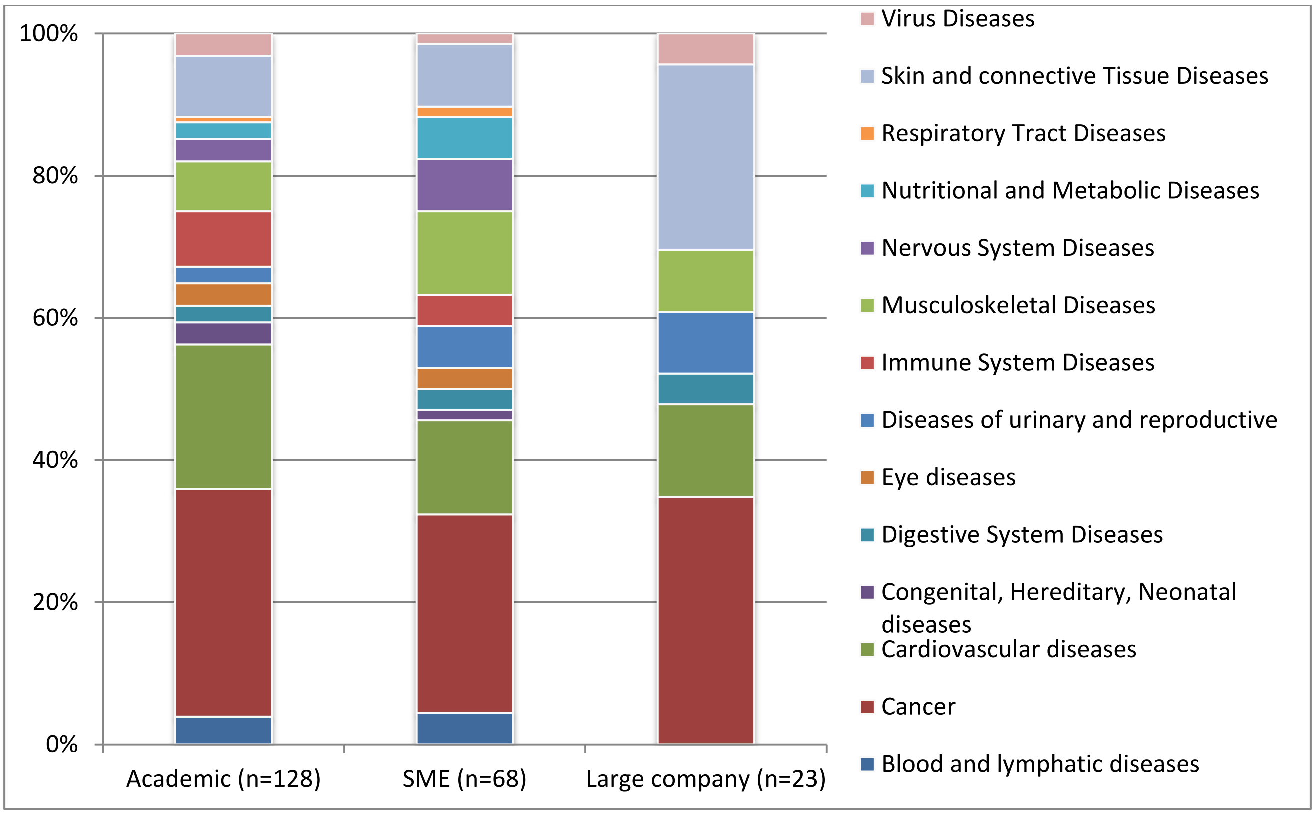

Figure S5. Indication area per subtype of gene and cell based therapies.

GTMP = gene therapy medicinal product; TEP = tissue-engineered product**.** Distribution of the different indication areas in which clinical trials were performed per product subtype.

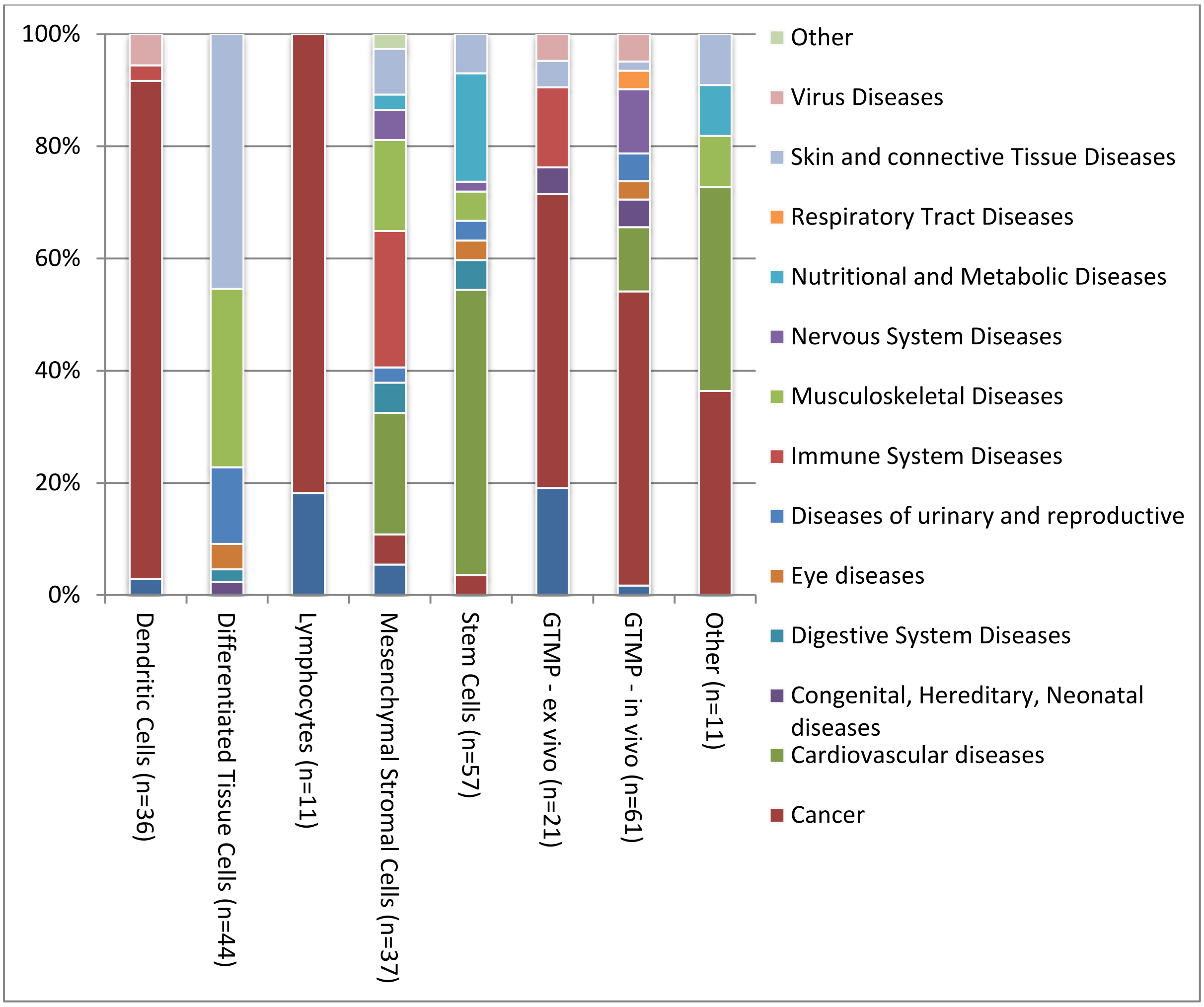

Figure S6. Gene and cell based therapy subtype per sponsor.

CTMP = cell therapy medicinal product; GTMP = gene therapy medicinal product; SME = small and medium-sized enterprise; TEP = tissue-engineered product. Distribution of the gene and cell based therapy product subtype per sponsor.

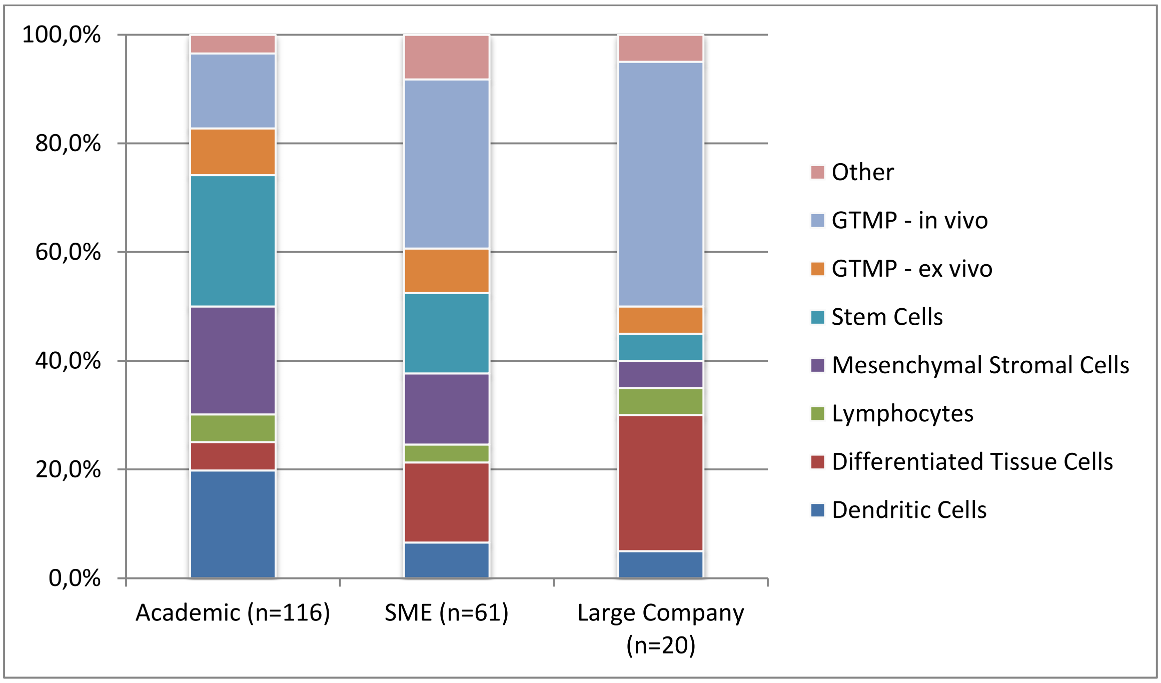

Figure S7. Starting material per sponsor.

SME = small and medium-sized enterprise. Distribution of autologous or allogeneic starting materials used for the gene and cell based therapy per sponsor.

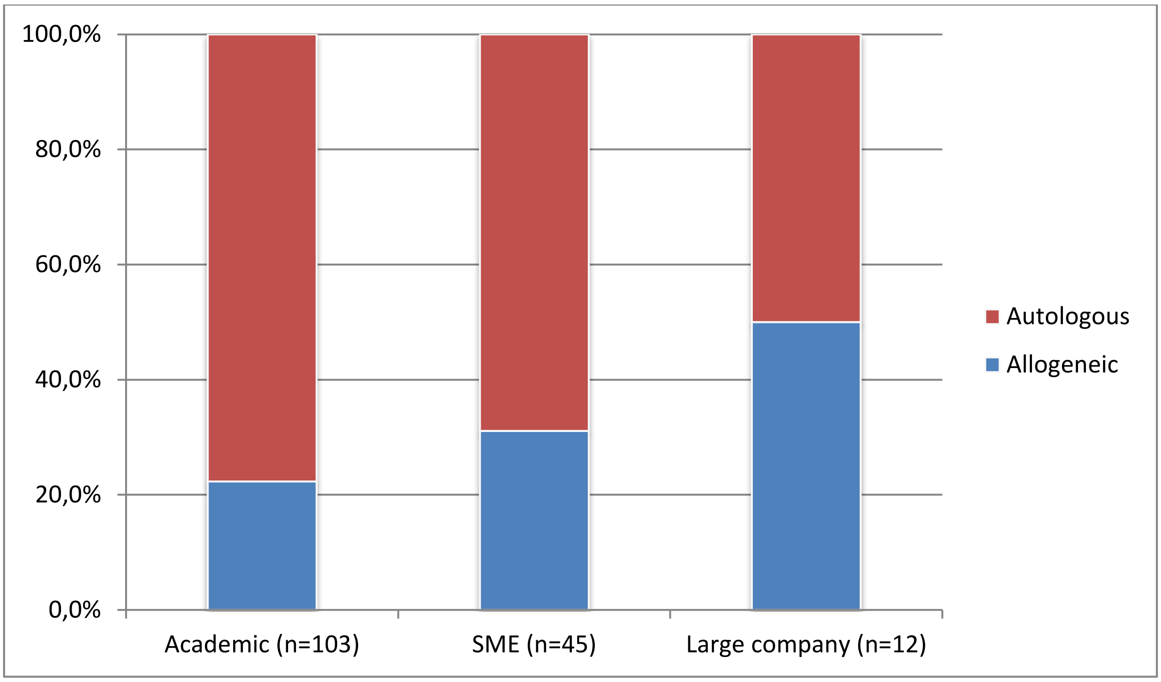

Figure S8. Gene and cell based therapy subtype per starting material.

Distribution of the gene and cell based therapies manufactured from autologous of allogeneic starting material.

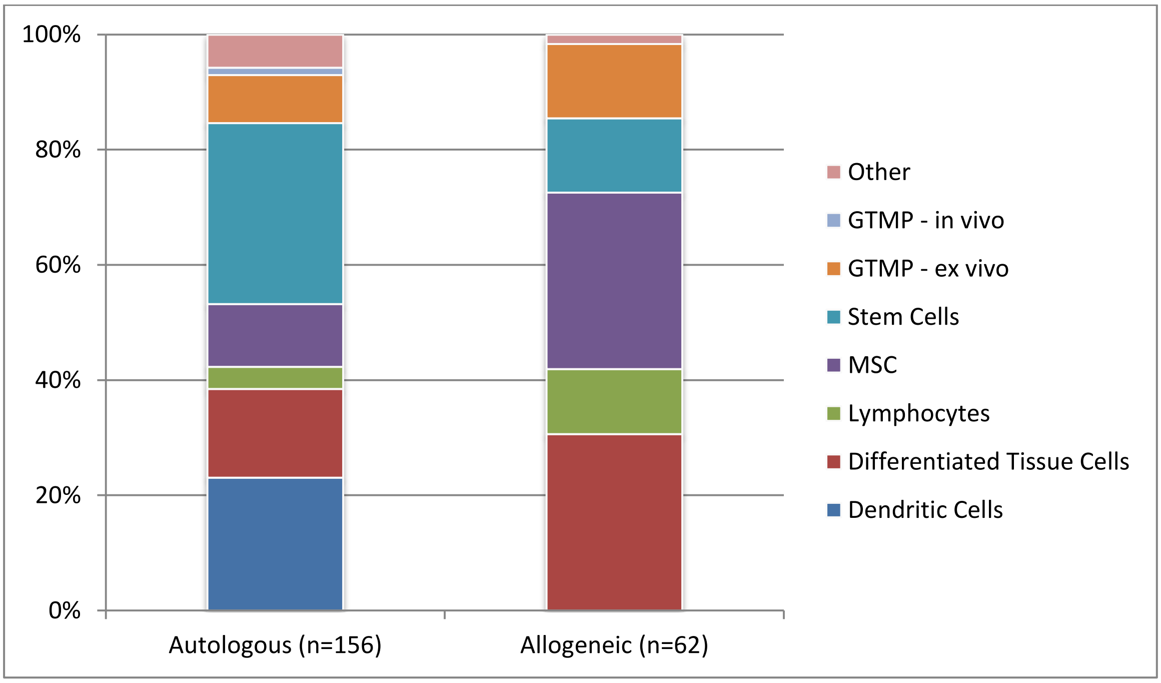

Figure S9. Starting material sources.

Distribution of the starting material sources per autologous or allogeneic derived starting material used for gene and cell based therapy.

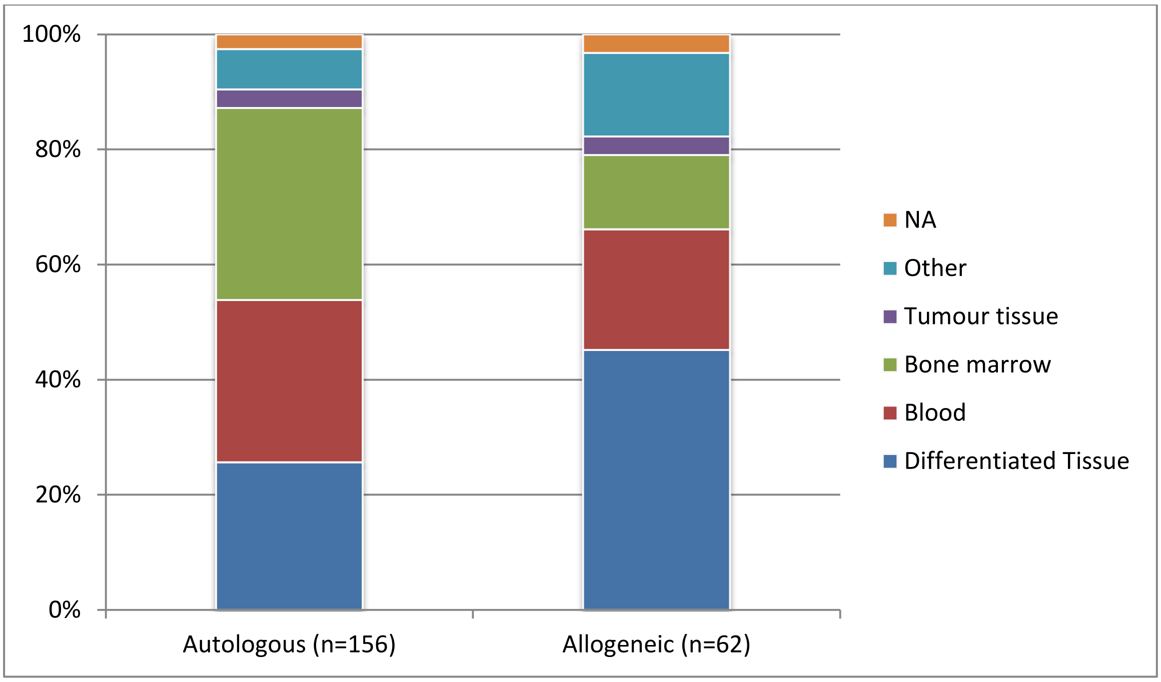

Figure S10. Starting material source per sponsor.

SME = small and medium-sized enterprise. Distribution of the starting materials sources which were used for gene and cell based therapies per sponsor type.

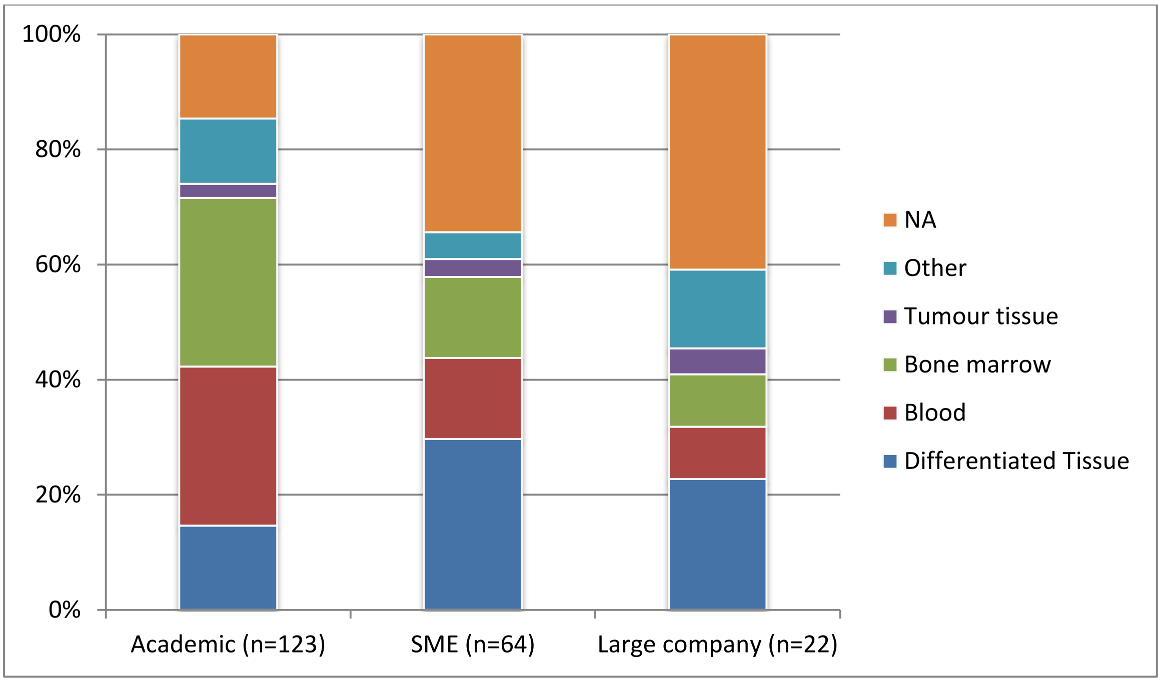

Figure S11. Indication area per starting material.

Distribution of the different indication areas in which clinical trial were performed with gene and cell bases therapy per autologous or allogeneic starting material.

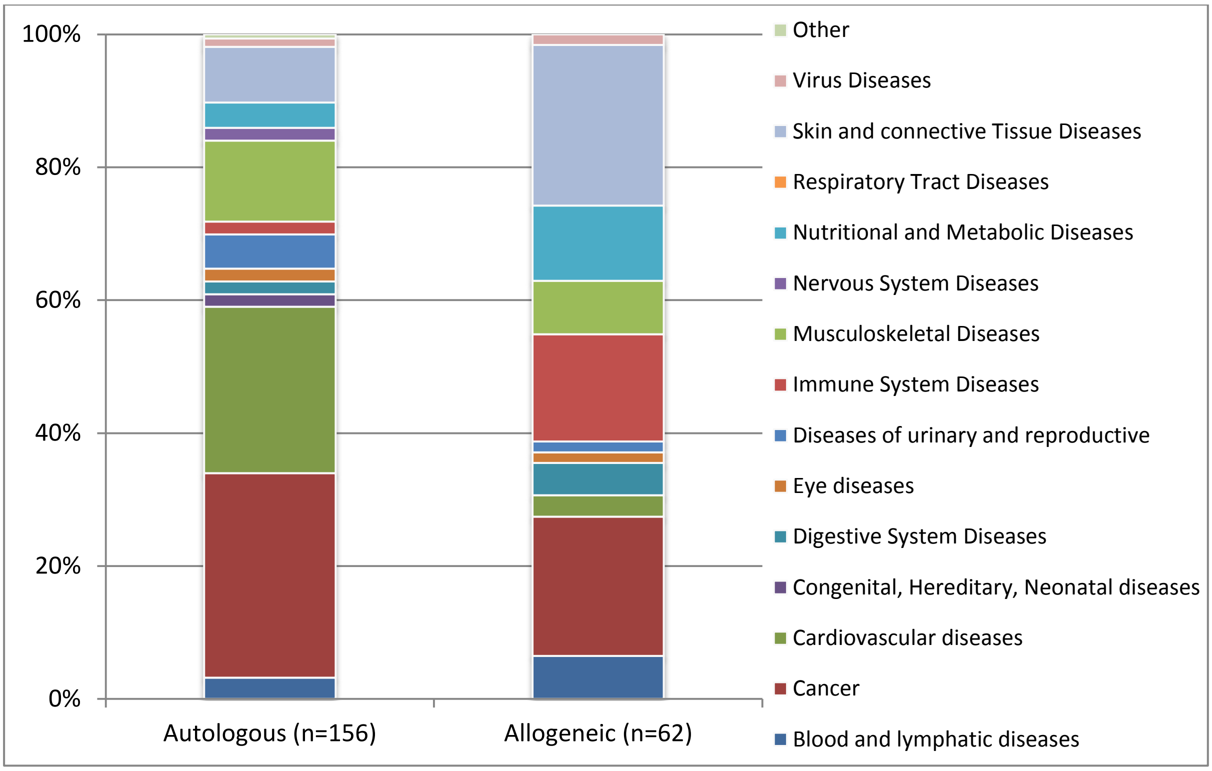

Figure S12. Indication area per starting material source.

Distribution of the different indication areas in which clinical trial were performed with gene and cell bases therapy per starting material source.

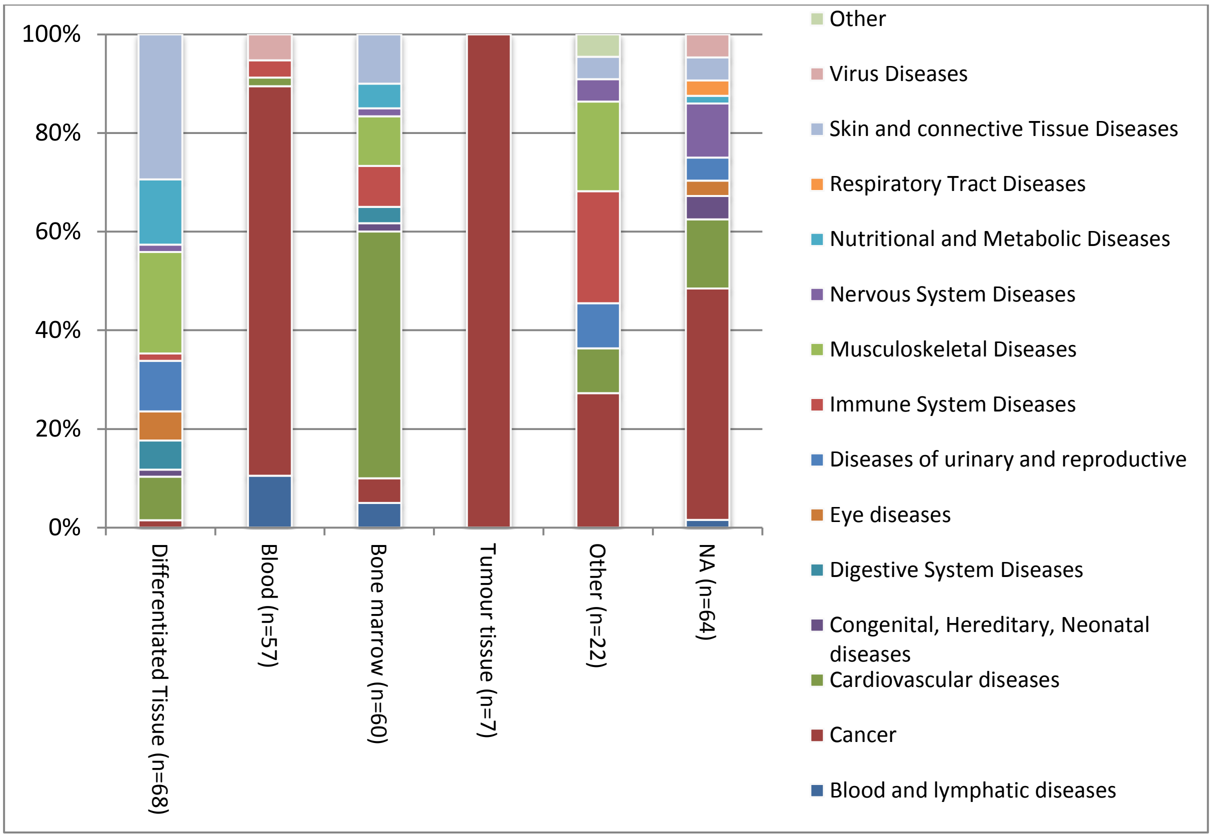

Supplement: Supplementary Figures [file mtm201673-s1.pdf]
